# Predicting range shifts of three endangered endemic plants of the Khorassan-Kopet Dagh floristic province under global change

**DOI:** 10.1038/s41598-021-88577-x

**Published:** 2021-04-28

**Authors:** Mohammad Bagher Erfanian, Mostafa Sagharyan, Farshid Memariani, Hamid Ejtehadi

**Affiliations:** 1 Quantitative Plant Ecology and Biodiversity Research Lab., Department of Biology, Faculty of Science, Ferdowsi University of Mashhad, PO BOX 9177948974, Mashhad, Iran; 2 Department of Plant Biology, Faculty of Biological Science, Tarbiat Modares University, Tehran, Iran; 3 Herbarium FUMH, Department of Botany, Research Center for Plant Sciences, Ferdowsi University of Mashhad, Mashhad, Iran

**Keywords:** Climate-change ecology, Biogeography

## Abstract

Endemic plants of the Khorassan-Kopet Dagh (KK) floristic province in northeastern Iran, southern Turkmenistan, and northwestern Afghanistan are often rare and range-restricted. Because of these ranges, plants in the KK are vulnerable to the effects of climate change. Species distribution modelling (SDM) can be used to assess the vulnerability of species under climate change. Here, we evaluated range size changes for three (critically) endangered endemic species that grow at various elevations (*Nepeta binaloudensis*, *Phlomoides binaludensis*, and *Euphorbia ferdowsiana*) using species distribution modelling. Using the HadGEM2-ES general circulation model and two Representative Concentration Pathways Scenarios (RCP 2.6 and RCP 8.5), we predicted potential current and future (2050 and 2070) suitable habitats for each species. The ensemble model of nine algorithms was used to perform this prediction. Our results indicate that while two of species investigated would benefit from range expansion in the future, *P*. *binaludensis* will experience range contraction. The range of *E*. *ferdowsiana* will remain limited to the Binalood mountains, but the other species will have suitable habitats in mountain ranges across the KK. Using management efforts (such as fencing) with a focus on providing elevational migration routes at local scales in the KK is necessary to conserve these species. Additionally, assisted migration among different mountains in the KK would be beneficial to conserve these plants. For *E. ferdowsiana*, genetic diversity storage employing seed banks and botanical garden preservation should be considered.

## Introduction

Climate is one of the main determinants delimiting geographical distribution of plant species on large scale^1^. There is a considerable amount of research declaring climate change lead to the range expansion or retraction in plant species ranges (e.g. Refs.^2–5^). To assess the vulnerability of plant species under a rapidly changing climate, we can use species distribution modelling (SDM) to predict species climate niches and project their potential future range shifts^4,6–8^. The SDM results can be used to develop adaptive management strategies, including assisted migration, to mitigate the effects of climate change^1,2,9^.

**Figure 1:**
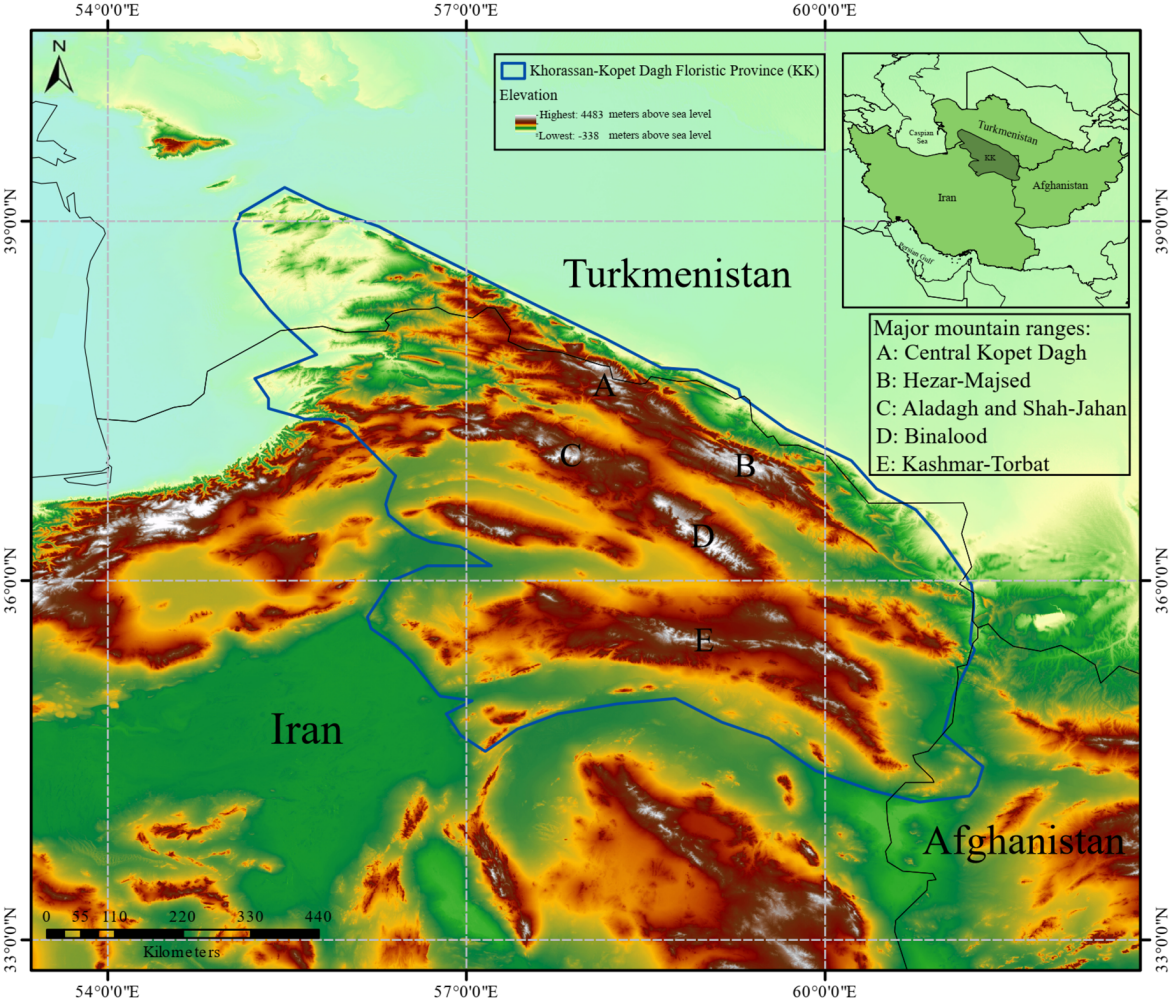
Study area—the Khorassan-Kopet Dagh floristic province (KK). The elevation range reported in the legend is for the entire map and not corresponds to the elevation range of KK. The original figure was adopted from Ref.^14^.

The Khorassan-Kopet Dagh (KK) floristic province is located mainly in northeastern Iran, and partially in southern Turkmenistan and northwestern Afghanistan (Fig. 1)^10–12^. This region is an under-studied biogeographic entity. A total number of 2576 vascular plant species have been recorded from this region. Among these, 356 species are endemics^13^. Except for a recent study on *Dianthus polylepis*^14^, no studies have been conducted to investigate the effects of climate change on endemic plants in the KK. *Nepeta binaloudensis* Jamzad (Lamiaceae) is a perennial species endemic to the KK. This plant grows in the elevation range of 2300–3000 m.a.s.l. in the Binalood Mountains. It has recently been recorded from the Hezar-Masjed Mountains^15–17^. *N*. *binaloudensis* is used in traditional medicine in northeastern Iran^18,19^. Antispasmodic, anti-allergic, and antidepressant effects have been reported for this plant. As a result, local people collect *N*. *binaloudensis* from the Binalood Mountain Range^17,19^. This species has been categorized as an endangered plant int the KK^20^. *Phlomoides binaludensis* Salmaki & Joharchi (Lamiaceae) is another endemic species to the KK. Hitherto, *P*. *binaludensis* has only been recorded from the Binalood Mountains. This perennial plant grows in the elevation range of 1350–2000 m.a.s.l.^21^. *P*. *binaludensis* has been categorized as an endangered plant^20^. *Euphorbia ferdowsiana* Pahlevani (Euphorbiaceae) is an endemic perennial growing in the Binalood Mountains. It has a narrow distribution range in the eastern slopes of this mountain range and has been recorded from the elevation range of 2100–2700 m.a.s.l.^22^. Due to its limited records, this plant has been categorized as a critically endangered species^20^.

Here, considering three [critically] endangered endemics that grow in the elevation range of 1350–3000 m.a.s.l. in the KK, we aimed to evaluate how climate change affects their potential distribution range. Specifically, our goals were to (a) model the current potential suitable habitats of these species; (b) study the effects of climate change on their current range size by using an ensemble model of nine modelling algorithms for 2050 and 2070 under the most optimistic and pessimistic climate change scenarios; (c) evaluate the future elevation shifts of these plants. We conducted this study to provide management guidance to protect endangered endemic plants from the effects of climate change in this poorly studied region.

## Materials and methods

### Study area

Our study area was the KK floristic province (Fig. 1). This region is a transitional zone connecting different phytogeographical units of the Middle East (i.e., Irano-Turanian region)^11^. According to Olson et al.^23^, six ecoregions (namely the Badghyz and Karabil semi-desert, Central persian desert basins, Elburz range forest steppe, Kopet Dag semi-desert, Kopet Dag woodlands and forest steppe, and Kuh Rud and Eastern Iran montane woodlands) are completely or partially located in the study area. The KK occupies an area of around 165,000 km^2^ and has a complex topography ranging from approximately 250 m.a.s.l. in the lower foothills to the elevations higher than 3000 m.a.s.l. in the Shirbad (3319 m.a.s.l.), Hezar-Masjed (3128 m.a.s.l.), and Shah-Jahan (3032 m.a.s.l.) Mountains^11,13^. The KK has a continental climate. Mountain ranges in KK have a Mediterranean or Irano-Turanian xeric-continental bioclimate with an average annual precipitation of 300–380 mm. The mean annual temperature in the area shows elevation-dependent values and ranges between 12–19 °C^11,24^. The KK is home to diverse vegetation types. Among these types, the montane steppes and grasslands are the most abundant. Furthermore, it is an important hotspot for other species groups^12,25,26^. The area is hosting 2576 species or infraspecific vascular plant taxa. Among these species, 356 (13.8 percent of the total species pool) are endemic to this region^11,13,20^. Most of the endemic species of the KK are range-restricted and rare^27^. The mountainous habitats in the KK are threatened by various disturbances (e.g. overgrazing, land use change, and recreation activities)^28,29^. Approximately, eight percent of the KK habitats are protected and managed with different protection guidelines^11,13,28–32^.

### Species data

Occurrence records for the studied species were obtained from botanical surveys, including the records from Ferdowsi University of Mashhad Herbarium (FUMH). These surveys were mostly one-time botanical surveys to collect plant specimens from different parts in the KK. Data collection were mainly carried out from 2002 to 2018. The occurrence points that entered in the analysis included all possible environmental conditions in that the studied species have been recorded from the KK. True absence points were collected from plot sampling and botanical surveys. Plot samplings have been conducted in some parts of the study region. Nevertheless, botanical surveys have been conducted in the entire area. For *N*. *binaloudensis*, we opted to use absence points from plots that were sampled from the Binalood and Hezar-Masjed Mountain Ranges. For the other two that are exclusive to the Binalood Mountains, we included the absence points from botanical surveys across mountain ranges outside of the Binalood Mountains. Furthermore, for these two species, true absences from the Binalood Mountains were obtained from plot data. Species occurrence points are included in Supplementary Information. Nevertheless, we included pseudoabsence (i.e., background) points in our modelling because the number of true absence points was low. The study area was defined based on biogeographical boundaries and absence points were randomly selected from all points within geographic range of the species. Three sets of background points (n = 10,000) were generated using the biomod2 package^33^. All calculations were performed in R ver. 3.6^34^.

We filtered occurrence data by randomly selecting a presence point within a single grid cell (i.e., 1 × 1 km) using the spThin package^35^. Finally, for *N*. *binaloudensis*, 13 occurrence points + 36 true absence points + three sets of 10,000 background points; for *P*. *binaludensis*, 11 occurrence points + 569 true absence points + three sets of 10,000 background points; and for *E*. *ferdowsiana*, 5 occurrence points + 580 true absence points + three sets of 10,000 background points were used for the modelling.

### Environmental data

We used physiographic maps and bioclimatic variables in SDM. Physiographic maps were: elevation, slope, and aspect with a 1-km^2^ resolution. In this study, 19 bioclimatic layers (Table 1), which are reliable in defining the physio-ecological tolerances of a species were used. These layers were downloaded from Worldclim^36^. Future layers for 2050 and 2070 were also downloaded. To download future layers, the Hadley Centre Global Environmental Model version 2‐Earth System (HadGEM2-ES) general circulation model (GCM) and two Representative Concentration Pathways (RCPs) scenarios [i.e., RCP 2.6 (most optimistic) and RCP 8.5 (most pessimistic)] were selected. We used the HadGEM2-ES model because it showed appropriate temperature forecasting when compared with the real data obtained from different synoptic stations in Iran^37^. The resolution of the current and future layers was 1-km^2^.

**Table 1:** Nineteen bioclimatic variables that were used to model the suitable habitats for the studied species in the present study.

Bioclimatic variable	Name
Mean annual temperature	BIO1
Mean Diurnal range	BIO2
Isothermality	BIO3
Temperature seasonality	BIO4
Maximum temperature of warmest month	BIO5
Minimum temperature of coldest month	BIO6
The annual temperature range (BIO5–BIO6)	BIO7
the mean temperature of wettest quarter	BIO8
the mean temperature of the driest quarter	BIO9
Mean temperature of warmest quarter	BIO10
Mean temperature of coldest quarter	BIO11
Annual precipitation	BIO12
Precipitation of wettest month	BIO13
Precipitation of driest month	BIO14
Precipitation seasonality (standard deviation/mean)	BIO15
Precipitation of wettest quarter	BIO16
Precipitation of driest quarter	BIO17
Precipitation of warmest quarter	BIO18
Precipitation of coldest quarter	BIO19

To select layers (i.e., physiographic and bioclimatic layers), we performed a principal component analysis (PCA) and visualized the correlation between the environmental layers. PCAs were performed in the ade4 package by using the *dudi.pca* function^38^. Layers with the most contribution in explaining the variation of the species occurrence points space were selected. As suggested by Guisan et al.^39^, this analysis was performed. To remove collinear layers, we calculated variance inflation factor (VIF) for the selected layers. VIFs were calculated by performing a step-by-step process using the usdm package^40^. We selected those variables with VIF below ten, because VIF above ten shows a serious collinearity problem^41^. A list of the selected variables for each species is presented in Supplementary Table S1 of supplementary data.

### Modelling settings

Nine modelling algorithms—including the Generalised Linear Model (GLM), Generalized Additive Model (GAM), Generalized Boosting Model (GBM), Classification Tree Analysis (CTA), Artificial Neural Network (ANN), Surface Range Envelop (SRE, also known as BIOCLIM), Multiple Adaptive Regression Splines (MARS), Random Forest (RF), and Maximum Entropy (MAXENT)—were used in this study. These nine algorithms are available in the biomod2 package. We randomly split the occurrence data into two subsets, 70 percent of the data were used for the model calibration. The remaining 30 percent was used for model evaluation. We split data because we did not have independent data for model evaluation. The number of replications was set to ten to calculate each model.

To measure SDM performance, we employed the True Skill Statistic (TSS) and Area Under Curve (AUC) of the Receiver Operation Curves. TSS is a threshold-dependent measure, ranges between − 1 and + 1, where + 1 indicates perfect agreement between predictions and observations, and values of 0 or less indicate agreement no better than random partitioning^42^. AUC is widely used to determine the predictive accuracy of SDMs. Generally, AUC ranges between 0.5 to 1.0 and models with AUC > 0.9 are categorized as very good^5^. For the binary transformation, we employed the threshold maximizes TSS to convert the occurrence probability values into presence/absence predictions. The thresholding approach that maximizes TSS is well suited because it produces the same threshold using either presence-absence or presence-only data^39^. These calculations were performed by using the biomod2 package^33^.

### Ensemble forecasting

We used the ensemble forecasting procedure to obtain final models in order to reduce the uncertainty among the species distribution algorithms. To combine models, we selected those with TSS > 0.9. Ensemble models were predicted for current and future conditions at a 1-km^2^ resolution. The ensemble models were converted into binary presence-absence predictions using the threshold that maximizes TSS. The ensemble forecasting was performed in the biomod2 package.

### Range and elevational shifts

To evaluate the range size changes of the studied species, we compared the predicted models for the future distribution of the species to that of their current distribution. Then, we distinguished four distinct habitat types; (a) stable habitats: habitats that are suitable in current and future climatic conditions; (b) lost habitats: currently suitable habitats that will not remain suitable in future; (c) gained habitats: currently unsuitable habitats that will become suitable in future; (d) unsuitable habitats: habitats that are unsuitable for species both in the current and future climatic conditions. Range size changes were predicted by using the biomod2 package^33^.

The predictive ensemble maps for the current and future (i.e., 2050 and 2070) were related with elevation classes. We extracted the elevation of the grids correspond to species presence from the current and future ensemble maps. Then, bar plots were drawn in R to compare the elevational range of the potential current and future habitats.

## Results

### Modelling evaluation

**Figure 2:**
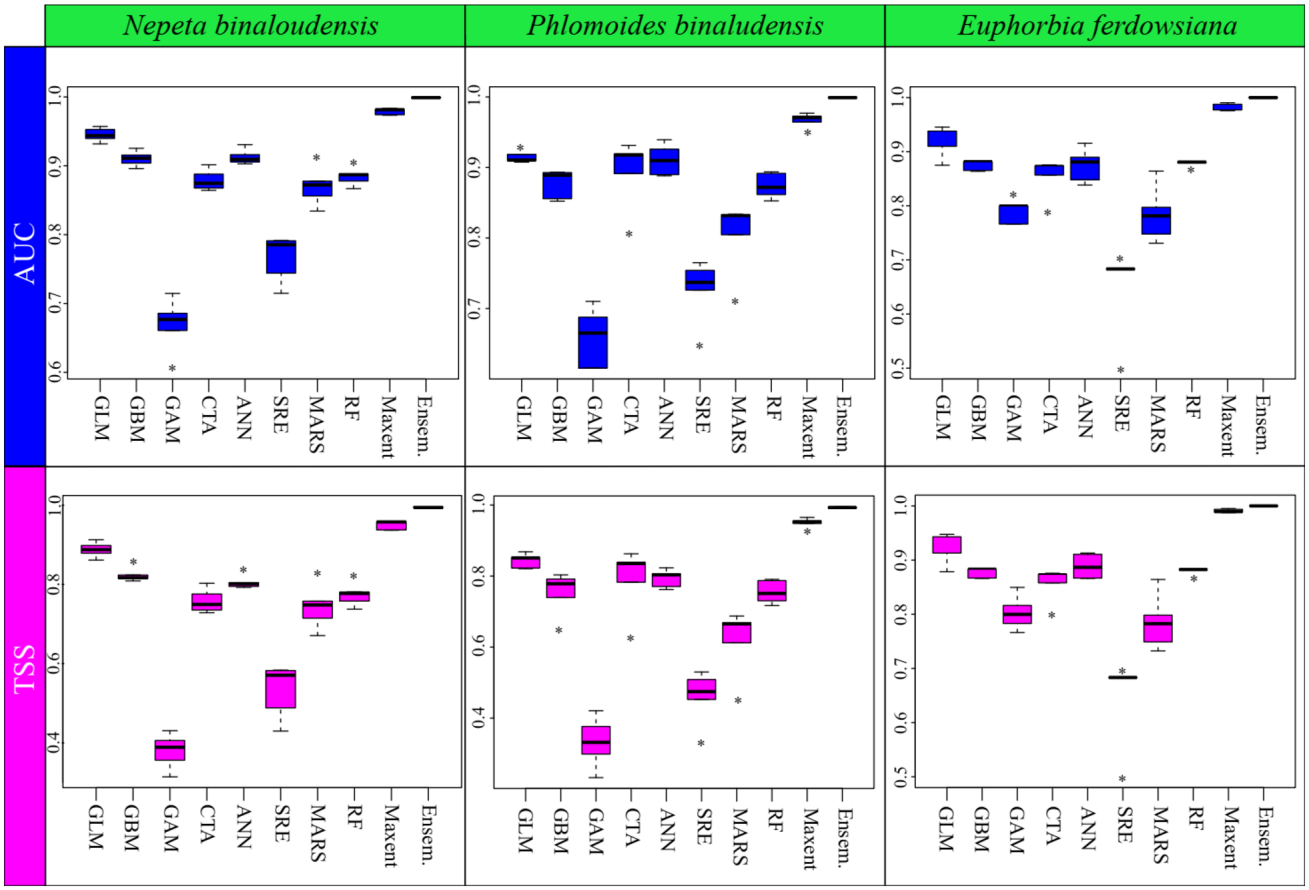
Boxplots showing the AUC and TSS scores for model evaluation from ten cross‐validation runs on test data for the nine SDM algorithms that were used for the prediction of three species distribution in the Khorassan-Kopet Dagh floristic province. For comparison, the evaluation scores for the ensemble model are shown. The ensemble model does not include those models with a TSS < 0.9. See the text for model abbreviations.

The MAXENT and GLM algorithms performed better than the other algorithms. In the studied species, the ensemble models had the best overall performance with both TSS and ROC values above 0.99. Figure 2 presents the predictive performance of the nine modelling algorithms for studied species showing inter-model variability.

### *Nepeta binaloudensis*

**Figure 3:**
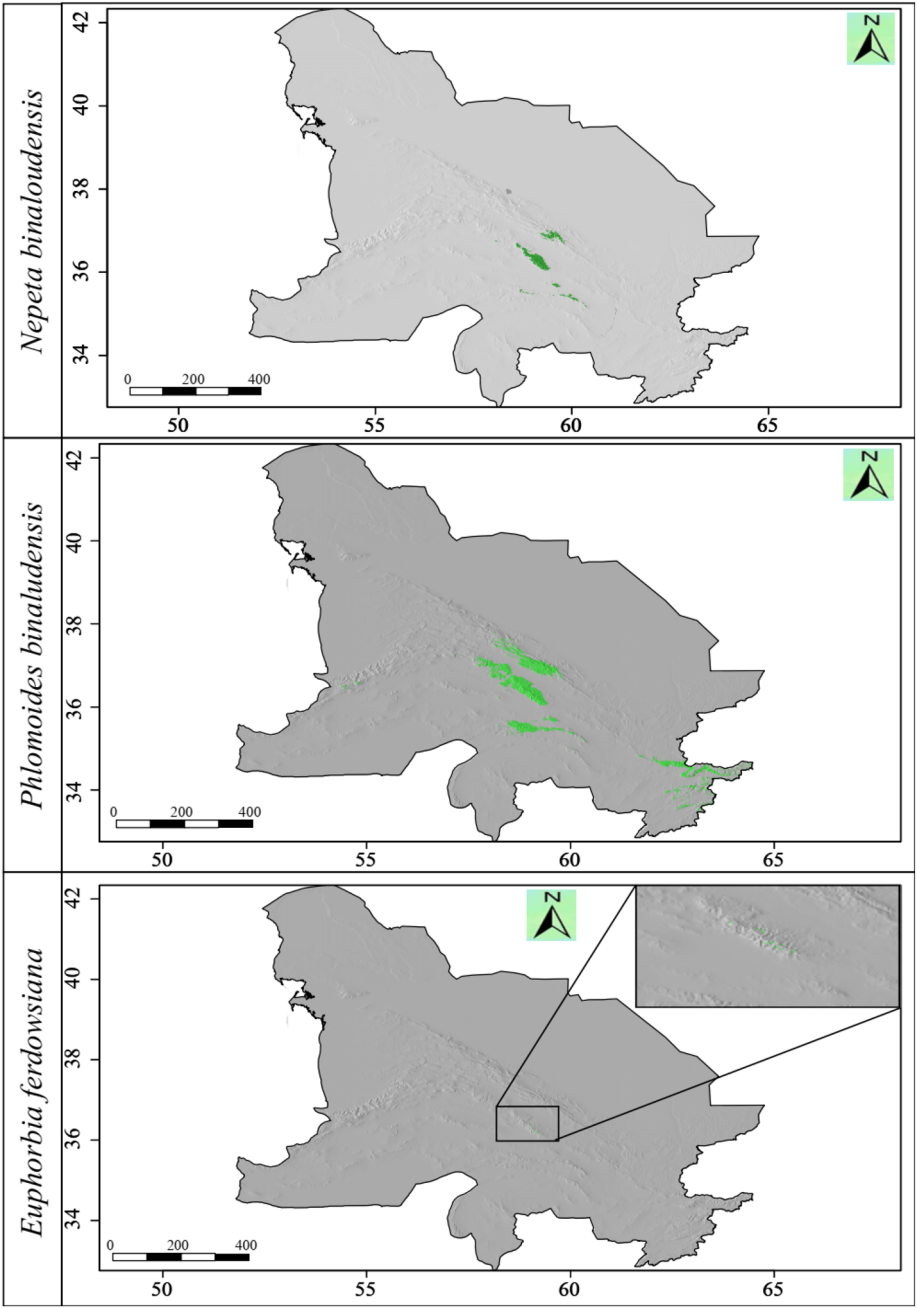
The habitat suitability maps of *Nepeta binaloudensis*, *Phlomoides binaludensis*, and *Euphorbia ferdowsiana* under the current climate conditions in the Khorassan-Kopet Dagh floristic province. Green areas indicate suitable habitats. The base map does not match that of KK boundaries and covers peripheral areas. The following maps have been generated in R ver. 3.6 (https://www.r-project.org).

**Figure 4:**
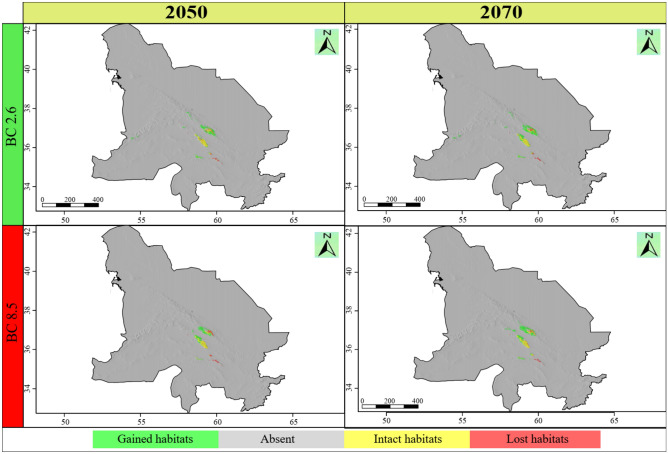
Range size changes of *Nepeta binaloudensis* in the future years (2050 and 2070) under the most optimistic (RCP 2.6) and pessimistic (RCP 8.5) scenarios. The base map does not match that of KK boundaries and covers peripheral areas. The following maps have been generated in R ver. 3.6 (https://www.r-project.org).

The ensemble habitat suitability map showed that the area of the currently suitable habitats for *N*. *binaloudensis* was 3407 km^2^ (Fig. 3). The base map that was used to show SDM results does not match that of the KK boundaries. This map also covers peripheral areas. The range size analysis showed that in 2050 and under RCP 2.6 (the most optimistic) scenario, 708 km^2^ of the currently suitable habitats will be lost, which is approximately 21 percent of the current habitats. Nevertheless, 3408 km^2^ would become newly suitable habitats that is equal to approximately 100 percent increase in the current range size. Finally, 2699 km^2^ of the current habitats will remain suitable for this species. The range size change for this species will be 79 percent. In 2050 and under RCP 8.5 (the most pessimistic) scenario, 963 km^2^ (28 percent) of the currently suitable habitats will become unsuitable; on the other hand, 2947 km^2^ will become suitable—86 percent increase of the current range size. Finally, 2444 km^2^ (72 percent) of the current habitats will remain suitable. As a result, the changes in the range size for this species will be 58 percent (Fig. 4).

In 2070 and under RCP 2.6 scenario, 771 km^2^ (23 percent) of the currently suitable habitats will become unsuitable, 2636 km^2^ (77 percent) will remain suitable, and 4333 km^2^ will be newly suitable habitats indicating 127 percent increase. The range size changes for this plant will be 104 percent. Under RCP 8.5 scenario in 2070, 680 km^2^ (20 percent) will be lost. The stable area covers 2727 km^2^ (80 percent), and 4096 km^2^ will become suitable for this species growth (i.e., 120 percent habitat gain) (Fig. 4). Therefore, the range size change will be 100 percent for this year and under this scenario (Table 2).

**Table 2:** The range changes for the studied species under climate change conditions in KK.

Species	Climate scenario	Total range size (km^2^)	Stable habitats		Lost habitats		Gained habitats		Range size change	
			km^2^	%	km^2^	%	km^2^	%	km^2^	%
*Nepeta binaloudensis*	Current	3407	*	*	*	*	*	*	*	*
	2050—RCP 2.6	6107	2699	79	708	21	3408	100	2700	79
	2050—RCP 8.5	5391	2444	72	963	28	2947	86	1984	58
	2070—RCP 2.6	6969	2636	77	771	23	4333	127	3562	104
	2070—RCP 8.5	6823	2727	80	680	20	4096	120	3416	100
*Phlomoides binaludensis*	Current	19,311	*	*	*	*	*	*	*	*
	2050—RCP 2.6	7019	5578	29	13,733	71	1441	7	12,292	− 64
	2050—RCP 8.5	5388	5185	27	14,126	73	203	1	13,923	− 72
	2070—RCP 2.6	9615	6738	35	12,573	65	2877	15	9696	− 50
	2070—RCP 8.5	6489	5419	28	13,892	72	1070	6	12,822	− 66
*Euphorbia ferdowsiana*	Current	0	*	*	*	*	*	*	*	*
	2050—RCP 2.6	42	13	22	47	78	29	48	18	− 30
	2050—RCP 8.5	129	21	35	39	65	108	180	69	115
	2070—RCP 2.6	117	24	40	36	60	93	155	57	95
	2070—RCP 8.5	91	16	27	44	73	75	125	31	52

**Figure 5:**
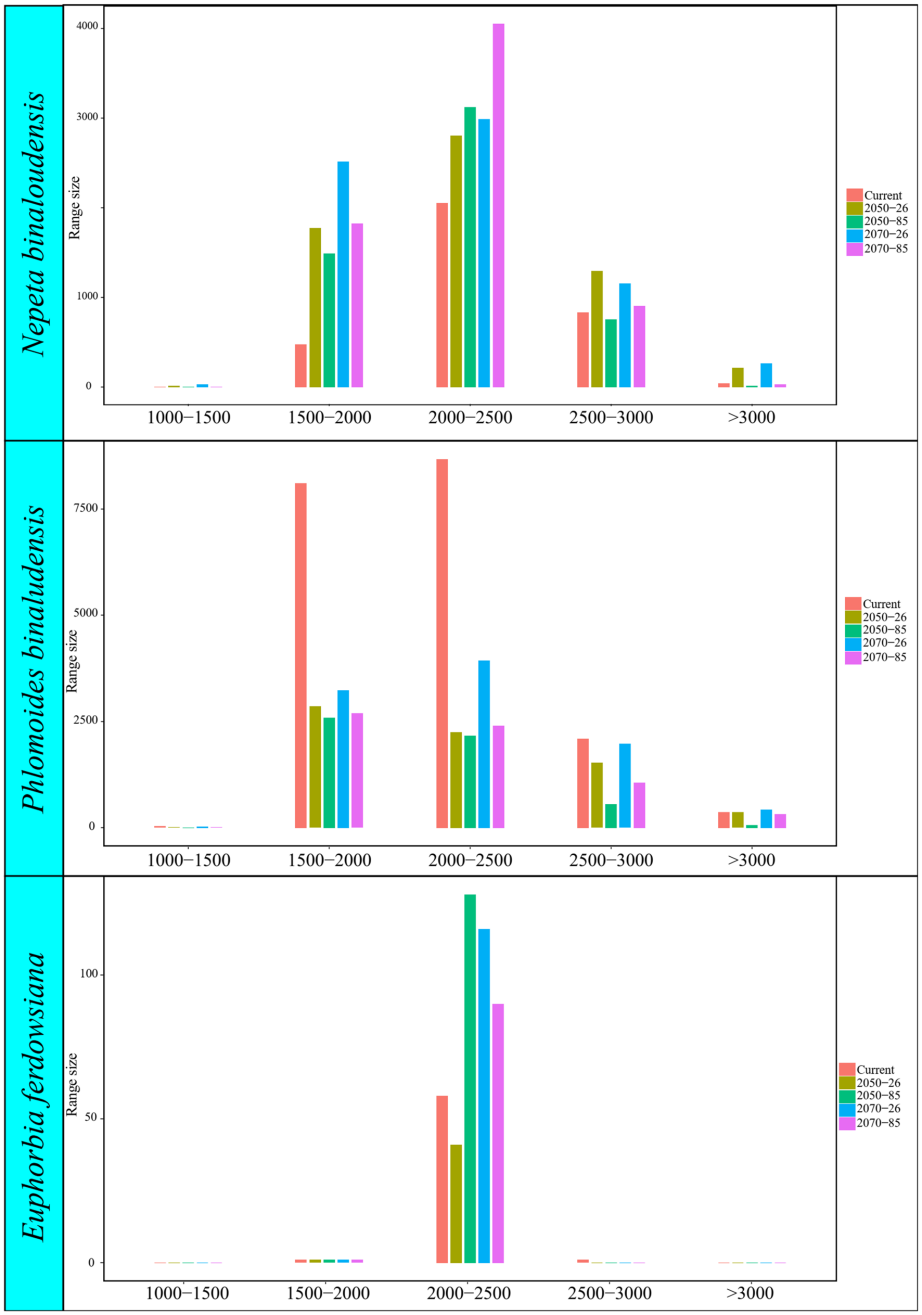
Range size of the studied species in relation to elevation classes under current and future climate change scenarios.

The comparison of ensemble maps along elevation classes indicated that *N*. *binaloudensis*, during both climate change scenarios, would expand its range in sites located between 1500–3000 m.a.s.l. In sites above 3000 m.a.s.l. depending on climate change scenario this plant would experience range expansion or contraction (Fig. 5).

### *Phlomoides binaludensis*

**Figure 6:**
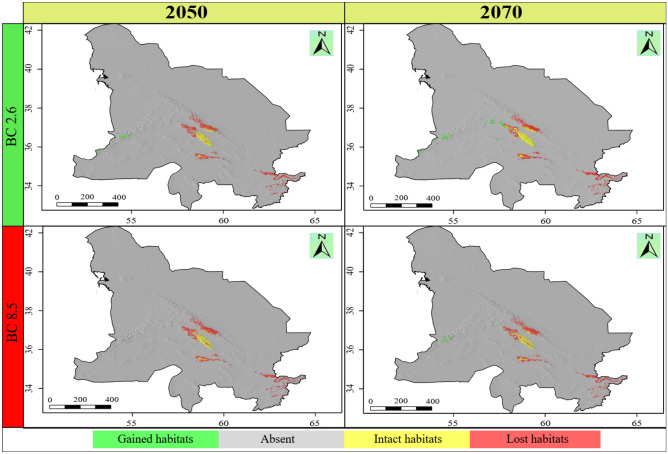
Range size changes of *Phlomoides binaludensis* in the future years (2050 and 2070) under most optimistic (RCP 2.6) and pessimistic (RCP 8.5) scenarios. The base map does not match that of KK boundaries and covers peripheral areas. The following maps have been generated in R ver. 3.6 (https://www.r-project.org).

The ensemble habitat suitability map showed that the current range size (i.e., potential current suitable habitats) for *P*. *binaludensis* was 19,311 km^2^ (Fig. 3). In 2050 and under RCP 2.6 scenario, 13,733 km^2^ (71 percent) of the currently suitable habitats will become unsuitable, 5578 km^2^ (29 percent) will remain suitable, and 1441 km^2^ will be newly suitable areas for this species—7 percent increase in distribution. The range size changes for this plant will be − 64 percent. Under RCP 8.5 scenario in 2050, 14,126 km^2^ (73 percent) will become unsuitable. The stable habitats cover 5185 km^2^ (27 percent), and 203 km^2^ will become suitable for this species growth—1 percent habitat gain. As a result, the range size changes for this species will be − 72 percent (Fig. 6).

In 2070, under RCP 2.6 scenario, 12,573 km^2^ (65 percent) of the current habitats will become unsuitable, on the other hand, 2877 km^2^ will become suitable for this species growth—15 percent habitat gain. Also, 6738 km^2^ (35 percent) of the current habitat size will remain suitable. As a result, changes in the range size for this species will be − 50 percent. In this year and under RCP 8.5 scenario, 13,892 km^2^ (72 percent) of the currently suitable habitats will become unsuitable, 5419 km^2^ (28 percent) will remain suitable, and 1070 km^2^ (i.e., 6 percent habitat gain) will be newly suitable habitats for this species (Fig. 6). The range size changes for this species will be − 66 percent (Table 2).

The comparison of ensemble maps along elevation classes revealed that *P*. *binaludensis* would contract in sites that located above 1500 m.a.s.l. during future climate change scenarios. Depending on climate change scenario the amount of range contraction will vary (Fig. 5).

### *Euphorbia ferdowsiana*

**Figure 7:**
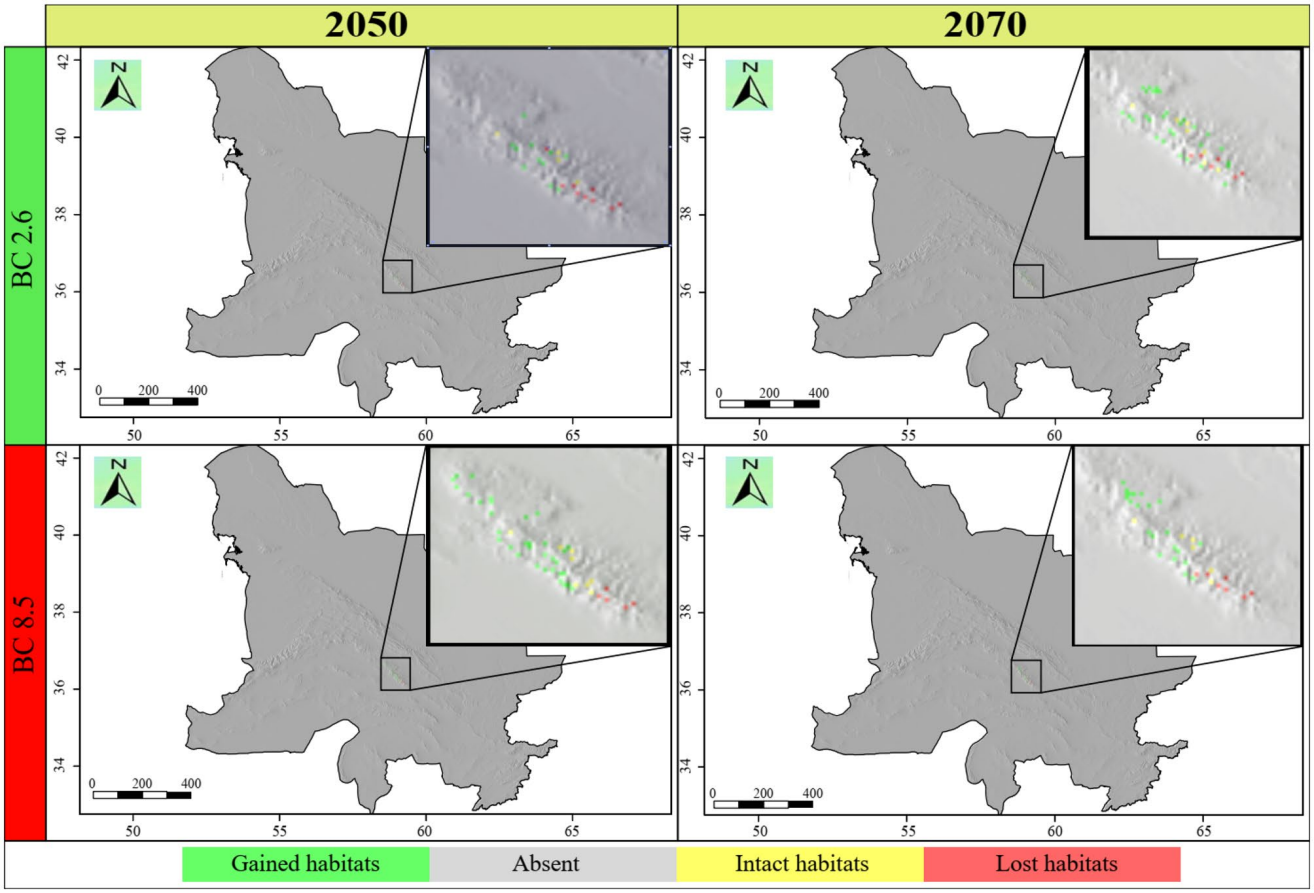
Range size changes of *Euphorbia ferdowsiana* in the future years (2050 and 2070) under most optimistic (RCP 2.6) and pessimistic (RCP 8.5) scenarios. The base map does not match that of KK boundaries and covers peripheral areas. The following maps have been generated in R ver. 3.6 (https://www.r-project.org).

The ensemble habitat suitability map showed that the current range size of *E*. *ferdowsiana* was 60 km^2^ (Fig. 3). In 2050 and under RCP 2.6 scenario, 47 km^2^ (78 percent) of the current suitable habitats will become unsuitable, 13 km^2^ (22 percent) will remain suitable, and 29 km^2^ (48 percent) will be newly suitable habitats for this species. Therefore, the range size changes for this species will be − 30 percent. Under RCP 8.5 scenario in 2050, 39 km^2^ (65 percent) will become unsuitable. The stable habitats will cover 21 km^2^ (35 percent), and 108 km^2^ will become suitable for this species growth—180 percent increase in distribution. As a result, the range size changes for this species will be 115 percent (Fig. 7).

In 2070, under RCP 2.6 scenario, 36 km^2^ (60 percent) of the current habitats will become unsuitable, on the other hand, 93 km^2^ (155 percent) will become suitable. Also, 24 km^2^ (40 percent) of the current suitable habitats will remain suitable. As a result, changes in the range size for this species will be 95 percent. In this year and under RCP 8.5 scenario, 44 km^2^ (73 percent) of the currently suitable habitats will be lost, 16 km^2^ (27 percent) will remain suitable, and 75 km^2^ will be newly suitable areas for this species (i.e., 125 percent habitat gain) (Fig. 7). The range size changes for *E*. *ferdowsiana* will be 52 percent (Table 2).

Considering distribution of *E*. *ferdowsiana* across elevation classes, a narrow elevation range of this plant was found. We compared future distribution across elevation classes and found that *E*. *ferdowsiana* would experience range contraction in sites between 2500 and 3000 m.a.s.l. Also, it would contract between 2000 and 2500 m.a.s.l. under RCP 2.6 in 2050 or it would experience range expansion considering the other studied climate change scenarios (Fig. 5).

## Discussion

### Model performance

In the current study, we showed that climate change will lead to species specific range expansion/contraction and elevational shifts. Our results revealed the variability among different SDM algorithms. Since there is no best algorithm for species distribution modelling, we used an ensemble model of the nine different algorithms. The ensemble model outperformed the other models. The efficiency of ensemble models was also reported by the other studies^6,43–45^. However, Hao et al.^46^ found no particular benefit in using ensembles over individual algorithms. Considering single algorithms, the MaxEnt showed the highest predictive performance. Kaky et al.^45^ suggested using the MaxEnt when computational power and knowledge is limited.

### Climate change impacts on *Nepeta binaloudensis*

The current potential suitable habitats of *N. binaloudensis* is largely located in the Binalood Mountains along with two patches in the Hezar-Masjed and Kashmar-Torbat Mountains. Ensemble map shows topographic-climatically suitable habitats in the Kashmar-Torbat Mountains. Hitherto, no literature data indicates the occurrence of this plant in this mountain range. Also, *N. binaloudensis* has not been found in floristic surveys in this area in the past ten years. This indicating a possible local extinction or lack of comprehensive floristic knowledge of the KK. A similar problem was reported for *Rosa arabica* in Egypt^47^. In 2050 and 2070 under the most optimistic scenario (RCP 2.6), a large part of the current suitable habitats that are located in the Binalood Mountains will remain unaffected. While range expansion will happen in the Hezar-Masjed Mountains in 2050 and 2070, the current suitable habitats in this mountain will become unsuitable. Translocation from lost habitats to gained habitats is necessary to ensure this species survival in the Hezar-Masjed Mountain Range. In 2050 and 2070, a northwestern migration trend will be observed for this species. As a result, new habitats will become suitable in the Central Kopet Dagh and Aladagh and Shah-Jahan Mountains. Under the most pessimistic scenario (RCP 8.5) in 2050 and 2070, a similar migration trend of RCP 2.6 will be detected. However, the Central Kopet Dagh Mountains will not become suitable habitat for this plant. As a result of climate change, a range expansion will be observed for this species. Range expansion as a result of climate change was reported for endemic herbs of Namibia^48^, some endemic plants of biodiversity hotspots in India^49^, plant species in Sardinia (Italy)^50^, various *Larix* species^51^, and some of European plant species^52^. While range expansion is supported by all scenarios in the lower elevation classes, in the higher elevations, this amplitude is not supported by all climate change scenarios (Fig. 5). Hitherto, there is no record of *N*. *binaloudensis* at elevations below 2000 m.a.s.l.^16,17,53^, the current distribution map indicated a possible local extinction in habitats located in this elevation range. This extinction can be due to extensive collecting of this medicinal plant^17^ or the long history of disturbances (e.g., land use changes and grazing) in the study area^28,29^.

### Climate change impacts on *Phlomoides binaludensis*

The potential current suitable habitats of this species are the Binalood Mountains, Hezar-Masjed, Aladagh and Shah-Jahan, Kashmar-Torbat, and some parts of northwestern Afghanistan mountains. Thus far, this species has only been recorded from the Binalood Mountains. This possible local extinction of potential suitable habitats might be due to the anthropogenic disturbances. Moreover, distribution range of this species might be limited by factors that were not included in this study (e.g., soil). We recommend further studies aiming to record *P*. *binaludensis* presence or investigate reasons for its absence in the potential suitable habitats of this plant that located outside the Binalood Mountain Range. Our results showed (Fig. 3), in the Binalood Mountains, this species has a broad distribution range that suggests its least vulnerability.

Due to the climate change, considering either scenarios or years, range contraction would be observed for this species. According to future distribution projections, a possible northwestern migration might occur. This could be a reason for confirming local extinction in its southern habitats—habitats that are climatically suitable without any recorded *P*. *binaludensis* occurrence. In the future, this plant will probably be less observed for the southern parts of the Binalood Mountain Range. Compared with *N*. *binaloudensis*, this species prefers cooler habitats. Due to climate change, suitable habitats for *P*. *binaludensis* will decrease, while *N*. *binaloudensis* experiences range expansion. Naturally, this plant will not migrate to the predicated gained habitats across the KK because of its limited range. Consequently, assisted migration should be used to introduce it to newly suitable habitats.

*Phlomoides* *binaludensis* has been recorded from elevation range of 1500–2500 m.a.s.l.^21^. Our results indicated massive range contraction in this elevation range (Fig. 5). However, range contraction at the upper elevations was not supported by all scenarios. Range contraction has been reported for many species in mountainous regions (e.g., Refs.^6,54–59^). As suggested by Abdelaal et al.^47^, the reason for range contraction is changing in the climatic envelope (precipitation and temperature).

### Climate change impacts on *Euphorbia ferdowsiana*

The current potential suitable habitats of *E*. *ferdowsiana* are restricted to the Binalood Mountain Range. The southern parts of this mountain range are the favourable habitats for this species. In 2050 and 2070, under either BC 2.6 or BC 8.5 current suitable habitats would become unfavourable. As a result, this species should migrate to the northwestern parts of the Binalood Mountain Range to ensure its survival. This critically endangered species will experience a limited range expansion within the Binalood Mountains. The results of the present study showed that this species has a narrow optimum elevational range (i.e., 2000–2500 m.a.s.l.). Due to climate change, no major elevation shift would occur for this plant.

### Limitations

We could not include soil, land use, and land cover variables for this study in the KK due to the fact that we did not have available data on a scale similar to bioclimatic variable (i.e., 1-km^2^). Successful migration of a species to potential future suitable habitats depends on climatic conditions, land use, biotic interactions, and the effects of microfugia^4^. Except for climatic conditions, we had no data on the other factors to assess the rate of successful future migrations. Adding seed dispersal ability as a variable in modelling plant range shifts resulted in a reduced number of possible future suitable habitats^60^, and this factor should be considered in interpreting the range expansion reported in this study.

## Conclusions

Here, we have determined three habitat types for the studied species. Proper conservation of these endangered species can be done based on what has been suggested by Fois et al.^61^. Monitoring, fencing, and reinforcement of the current populations are essential for intact habitats. For future suitable habitats (i.e., gained habitats), seeds and other propagules can be translocated from population in lost habitats. In the present study, the Dowlat Abad area located in central parts of the Binalood Mountain Range is a suitable area in order to protect the intact habitats. All studied plants have been recorded from the Dowlat Abad. For *E. ferdowsiana*, genetic diversity storage employing seed banks and botanical garden preservation should be considered.

We have studied three endangered plants that grow in different elevational ranges of KK mountains. The studied species will migrate to northwestern parts of the KK. Our results have showed that beside range contraction endangered species could also gain suitable future habitats. Our results can be used to propose proper management and conservation of the endangered species in the under-studied KK region. To decrease the risk of extinction in the wild, ex-situ and in-situ conservation activities for studied species are urgent. We suggest reinforcements of the existing populations in the Binalood Mountains and fencing different parts of this relatively disturbed mountain range. Also, programs of assisted migrations for *P*. *binaludensis* and *E*. *ferdowsiana* should be planned in the wild. No conservation program will be successful without increasing of public awareness and reducing the anthropogenic disturbances.

## Supplementary Information

Supplementary Information 1.

Supplementary Information 2.

## Data Availability

The data regarding this study are presented in Supplementary Information.
