# Supplementary material for: Predicting range shifts of three endangered endemic plants of the Khorassan-Kopet Dagh floristic province under global change
Source: Sci Rep. 2021 Apr 28;11:9159. doi: 10.1038/s41598-021-88577-x (PMC8080812; doi:10.1038/s41598-021-88577-x)
Supplement: Supplementary file 1 — Supplementary Information 1. [file 41598_2021_88577_MOESM1_ESM.pdf]

Predicting range shifts of three endangered endemic plants of the Khorassan-Kopet Dagh floristic province under global change

Mohammad Bagher Erfanian[1,*], Mostafa Sagharyan[2], Farshid Memariani[3], and Hamid Ejtehadi[1]

[1] *Quantitative Plant Ecology and Biodiversity Research Lab., Department of Biology, Faculty of Science, Ferdowsi University of Mashhad, Mashhad, Iran*

[2] *Department of Plant Biology, Faculty of Biological Science, Tarbiat Modares University, Tehran, Iran*

[3] *Herbarium FUMH, Department of Botany, Research Center for Plant Sciences, Ferdowsi University of Mashhad, Mashhad, Iran*

[*]Corresponding author: M.B. Erfanian, b.erfanian@um.ac.ir, Tel.: +98-51-38804167, Fax: +98-51-38796416, PO BOX 9177948974

decimalLongitude	decimalLatitude	Nepeta.binaloudensis
58.60859	36.6682	1
59.04881	36.32588	1
59.12677	36.28207	1
59.24686	36.21438	1
59.39079	36.99671	1
59.3987	37.00781	1
59.36183	36.97937	0
59.33874	36.9723	0
59.35057	36.96979	0
59.29109	36.95311	0
59.01947	36.83692	0
59.10613	36.33733	0
59.12553	36.38155	0
59.15599	36.42008	0
59.16349	36.43053	0
59.18635	36.44905	0
59.1991	36.4607	0
59.21352	36.46974	0
59.22557	36.48154	0
59.22229	36.49335	0
59.24055	36.50963	0
59.25917	36.52032	0
59.28256	36.53247	0
59.21331	36.53309	0
59.05428	36.28736	0
59.493	36.28445	0
59.07882	36.3053	0
59.11563	36.35953	0
59.13639	36.16472	0
59.47917	36.28861	0
59.49681	36.27522	0
59.53111	36.31196	0
59.5	35.5	0
60	36.316	0
63.30629	33.8855	0
55.09597	36.76064	0
62.45447	33.95406	0
57.67678	35.19537	0
63.03572	33.49345	0
57.69889	35.87946	0
64.36246	34.59253	0
54.57556	36.39169	0
59.184	36.13	1
59.282	36.249	1

59.108	36.282	1
59.206	36.306	1
59.131	36.312	1
59.108	36.371	1
59.284	36.428	1

decimalLatitude	decimalLongitude	Phlomoides.binaloudensis
36.087	59.288	1
36.121	59.37	1
36.274	59.116	1
36.305	59.155	1
36.354	58.889	1
36.377	59.258	1
36.584	58.949	1
36.59657	58.59321	1
36.78083	58.55241	1
36.36046	59.11382	1
36.3639	59.11003	1
36.72587	59.84027	0
36.68079	59.92821	0
36.69714	59.83772	0
36.6933	59.79366	0
36.69085	59.77002	0
36.75945	59.87055	0
36.7865	59.88192	0
36.78007	59.84174	0
36.74736	59.77523	0
36.77661	59.80681	0
36.75023	59.74981	0
36.66066	59.61685	0
36.69443	59.63573	0
36.80957	59.85363	0
36.79735	59.80603	0
36.78855	59.71586	0
36.78053	59.64075	0
36.76374	59.60461	0
36.75225	59.5694	0
36.70963	59.51126	0
36.73656	59.54857	0
36.74391	59.55933	0
36.76672	59.56239	0
36.84574	59.59843	0
36.83167	59.57944	0
36.90834	59.74107	0
36.88123	59.69605	0
36.86461	59.6513	0
36.85712	59.62217	0
36.81668	59.54302	0
36.81346	59.4931	0
36.96459	59.71897	0
36.91806	59.6534	0

36.90367	59.61714	0
36.89321	59.5878	0
36.87908	59.55394	0
36.8637	59.53741	0
36.84516	59.52282	0
36.82904	59.50353	0
36.80304	59.48163	0
36.77382	59.42939	0
36.80013	59.45515	0
36.82079	59.48514	0
36.84604	59.50428	0
36.88925	59.55656	0
36.91446	59.57303	0
36.93449	59.60411	0
36.93781	59.54898	0
36.91781	59.52216	0
36.9052	59.49514	0
36.89924	59.48142	0
36.89694	59.46765	0
36.87993	59.44947	0
36.86421	59.43076	0
36.85082	59.4125	0
36.82932	59.39336	0
36.83251	59.36401	0
36.85704	59.37502	0
36.87798	59.40154	0
36.88912	59.41974	0
36.90673	59.42252	0
36.92152	59.44999	0
36.93699	59.45983	0
36.97345	59.48022	0
36.99489	59.51211	0
37.02616	59.53403	0
36.99658	59.47834	0
36.97833	59.42321	0
36.97149	59.40169	0
36.95078	59.38326	0
36.93573	59.35447	0
36.91037	59.3211	0
36.90191	59.31593	0
36.92895	59.32427	0
36.84723	59.28712	0
36.89299	59.30308	0
36.93543	59.31138	0
37.00958	59.38496	0

37.04352	59.41232	0
37.03285	59.37978	0
37.00578	59.32974	0
37.00474	59.28308	0
37.02931	59.30033	0
37.03428	59.24338	0
37.07683	59.1004	0
36.9933	59.01839	0
37.05623	59.05242	0
37.05192	58.97054	0
36.97395	58.78465	0
37.03526	58.78311	0
37.09076	58.71078	0
36.71629	60.23407	0
36.71048	60.18659	0
36.69877	60.12353	0
36.67315	60.02527	0
36.65667	59.96075	0
36.63567	59.91268	0
36.57297	59.84025	0
36.52198	59.76838	0
36.47429	59.64499	0
36.45121	59.5874	0
36.38078	59.63273	0
36.38885	59.76544	0
36.31551	59.77293	0
36.39125	59.63537	0
36.78287	59.9055	0
36.84537	59.86076	0
36.84802	59.79867	0
36.834	59.75032	0
36.68648	59.67176	0
36.97482	59.64401	0
36.96569	59.61726	0
36.9428	59.58962	0
36.92633	59.57685	0
36.75098	59.00311	0
36.72546	58.99801	0
36.69726	59.0388	0
36.6704	59.04602	0
36.63369	59.06737	0
36.60689	59.08867	0
36.60017	59.27708	0
36.50842	59.46393	0
36.57348	60.02637	0

36.62673	60.04379	0
36.63979	59.957	0
36.64006	59.90089	0
36.69205	59.9505	0
36.70589	60.06947	0
36.74097	60.1353	0
36.78801	59.89403	0
36.79279	59.83774	0
36.78842	59.7974	0
36.77461	59.67137	0
36.80672	59.65334	0
36.76058	59.63706	0
36.72617	59.61535	0
36.71188	59.60217	0
36.69208	59.5939	0
36.75444	59.61189	0
36.76461	59.51963	0
36.74641	59.46525	0
36.73929	59.41904	0
36.74317	59.37281	0
36.70056	59.32138	0
36.68304	59.29178	0
36.64842	59.3232	0
37.10519	58.66687	0
37.16153	58.74415	0
37.39725	58.75718	0
37.42973	58.76681	0
37.39064	58.7024	0
37.36887	58.72768	0
37.40603	58.80474	0
37.42279	58.85227	0
37.3962	58.8224	0
37.35538	58.79043	0
37.34913	58.7529	0
37.3335	58.79561	0
37.28931	58.7534	0
37.2315	58.74865	0
37.17178	58.78164	0
37.18623	58.89712	0
37.24655	58.96168	0
37.18451	58.93679	0
37.23935	59.12079	0
37.22348	59.20629	0
37.17744	59.21417	0
37.10345	59.18024	0

37.06172	59.13421	0
37.10752	59.30888	0
37.10749	59.38991	0
36.87114	59.00148	0
36.94362	59.11303	0
36.89063	59.04438	0
36.95862	59.34409	0
36.97345	59.56467	0
36.91957	59.62678	0
36.85793	59.6341	0
36.77211	59.70606	0
36.74705	59.90592	0
36.64895	59.88526	0
37.06202	59.51236	0
37.08206	59.72071	0
37.09632	59.68558	0
36.87275	59.94283	0
36.84491	59.90472	0
36.74312	59.83154	0
36.68538	59.78646	0
36.63545	59.74637	0
36.65659	59.72264	0
36.63778	60.00589	0
36.63992	60.06291	0
36.6188	60.05853	0
36.58251	60.06474	0
36.35421	59.95143	0
36.41766	59.79367	0
36.63132	59.94818	0
36.64742	60.13899	0
35.91736	59.90191	0
37.13383	59.18624	0
37.11954	59.17257	0
37.12809	59.24688	0
37.10541	59.23697	0
37.07331	59.24598	0
37.13301	59.28483	0
37.10573	59.33741	0
37.08276	59.39067	0
37.05193	59.4345	0
37.03696	59.4313	0
37.0189	59.42701	0
37.01009	59.41643	0
36.99706	59.41325	0
36.98784	59.42173	0

37.01316	59.46277	0
37.04119	59.48855	0
37.0488	59.52081	0
37.03964	59.54007	0
37.0267	59.55947	0
37.58387	58.50859	0
37.58718	58.4829	0
37.59046	58.46525	0
37.61098	58.45875	0
37.6278	58.44982	0
37.6236	58.4209	0
37.62893	58.40728	0
37.63828	58.40899	0
37.6429	58.42152	0
37.63547	58.43907	0
37.64031	58.46109	0
37.63324	58.46883	0
37.62705	58.49811	0
37.56229	58.5277	0
37.55375	58.51759	0
37.54309	58.52863	0
37.51992	58.50506	0
37.50339	58.50401	0
37.49213	58.49729	0
37.47556	58.51304	0
37.48657	58.56216	0
37.46334	58.52748	0
37.4475	58.54779	0
37.44398	58.57374	0
37.46805	58.60307	0
37.49332	58.62248	0
37.5142	58.65011	0
37.57022	58.64852	0
37.58447	58.6235	0
37.57974	58.64947	0
37.59351	58.6549	0
37.56914	58.67166	0
37.5553	58.6723	0
37.53933	58.66175	0
37.52176	58.66365	0
37.50316	58.66087	0
37.48358	58.64729	0
37.46868	58.64103	0
37.45845	58.62539	0
37.44518	58.61833	0

37.43593	58.61947	0
37.43367	58.63367	0
37.44385	58.66118	0
37.43381	58.68178	0
37.44496	58.70646	0
37.46636	58.7246	0
37.46565	58.7445	0
37.47395	58.76965	0
37.48082	58.78849	0
37.47879	58.80368	0
37.47429	58.81453	0
37.4612	58.81204	0
37.4419	58.81486	0
37.42987	58.81314	0
37.41757	58.80764	0
37.40642	58.79225	0
37.3968	58.78119	0
37.38725	58.78461	0
37.37434	58.78114	0
37.36193	58.78201	0
37.35337	58.7765	0
37.33344	58.77286	0
37.31762	58.76875	0
37.3056	58.76627	0
37.29588	58.76719	0
37.28593	58.77233	0
37.28059	58.79659	0
37.27048	58.78714	0
37.27333	58.80388	0
37.27126	58.82509	0
37.26279	58.82949	0
37.25796	58.83984	0
37.27524	58.87566	0
37.29247	58.90336	0
37.2905	58.93204	0
37.31318	58.99175	0
37.30367	58.99031	0
37.28865	58.99512	0
37.27169	58.99948	0
37.25487	58.99908	0
37.28125	59.03889	0
37.26863	59.03595	0
37.2791	59.07253	0
37.26085	59.07753	0
37.24458	59.07515	0

37.22337	59.07305	0
37.19895	59.03243	0
37.23969	59.08601	0
37.2451	59.10678	0
37.22358	59.11034	0
37.20734	59.12431	0
37.19077	59.12379	0
37.16716	59.10816	0
37.14709	59.08275	0
37.12363	59.0788	0
37.09724	59.14135	0
37.15932	59.22035	0
36.98804	59.36854	0
36.97724	59.34903	0
36.96822	59.35293	0
36.97573	59.36268	0
36.97308	59.37416	0
36.96393	59.37963	0
36.97198	59.38929	0
36.95978	59.39166	0
36.9489	59.46336	0
36.94674	59.52436	0
36.90193	59.52708	0
36.86525	59.51955	0
36.84431	59.4805	0
36.83997	59.40608	0
36.80984	59.39116	0
36.77979	59.36932	0
36.78391	59.44785	0
36.79904	59.51969	0
36.81921	59.56883	0
36.83278	59.61224	0
36.86601	59.66622	0
36.89855	59.73299	0
36.90942	59.70175	0
36.87875	59.73941	0
36.86591	59.75986	0
36.85629	59.77853	0
36.83315	59.76618	0
36.805	59.75915	0
36.78632	59.75588	0
36.76819	59.75624	0
36.73565	59.7701	0
36.70669	59.7554	0
36.6754	59.7704	0

36.66928	59.74067	0
36.69311	59.98263	0
36.71901	60.01837	0
36.72071	59.81109	0
36.69962	59.77564	0
36.66905	59.80517	0
36.6202	59.75571	0
36.41803	60.19444	0
36.46594	60.23577	0
36.47342	60.10972	0
36.50448	60.07126	0
36.51629	60.09728	0
36.21894	60.23183	0
36.67597	59.88319	0
36.7147	59.86797	0
36.78502	59.86394	0
36.51202	59.76094	0
36.46622	59.81054	0
36.40607	59.84376	0
36.31713	59.85008	0
36.26437	59.92923	0
36.29456	59.6812	0
36.36691	59.59197	0
36.45213	59.54145	0
36.54269	59.40892	0
36.53261	59.3147	0
36.54833	59.24238	0
37.02681	58.74645	0
36.98371	58.65318	0
36.96409	58.56462	0
37.08635	58.52685	0
37.1551	58.58764	0
37.52053	58.58357	0
37.5915	58.58622	0
37.48382	58.71888	0
37.72075	58.31453	0
37.68569	58.24729	0
37.64897	58.30177	0
37.61491	58.37028	0
37.54925	58.43399	0
37.4854	58.48926	0
37.33888	58.84272	0
37.24313	58.99892	0
37.14985	58.97217	0
37.69238	58.43288	0

37.76501	58.26385	0
37.80498	58.14663	0
37.47866	58.175	0
36.60172	64.29742	0
34.46007	63.91618	0
34.45606	63.62844	0
34.4866	63.38501	0
34.4599	63.10192	0
34.44382	62.89625	0
34.62113	62.58306	0
34.63376	62.37054	0
33.8914	62.6445	0
33.63138	62.63398	0
33.43861	63.03738	0
34.86632	60.1907	0
35.04577	59.89041	0
35.51433	59.84767	0
35.38658	58.65703	0
33.35841	58.48966	0
35.63335	58.52979	0
35.52552	58.77781	0
36.58241	54.35698	0
36.63097	54.66044	0
36.66189	54.86709	0
36.76782	55.20019	0
37.05097	55.70175	0
37.37109	56.09588	0
37.45043	56.41716	0
38.80722	57.7982	0
37.82522	58.07355	0
37.35539	54.4676	0
37.39443	56.73825	0
35.4077	58.27722	0
35.36968	58.51215	0
35.3624	58.52906	0
35.34902	58.54431	0
35.33078	58.54551	0
35.31905	58.5733	0
35.33318	58.60281	0
35.34986	58.61669	0
35.36353	58.56574	0
35.36217	58.65041	0
35.34526	58.64843	0
35.33326	58.6522	0
35.35794	58.75499	0

35.33701	58.8087	0
35.36717	58.72784	0
35.37657	58.67398	0
35.37077	58.58624	0
35.35738	58.48484	0
35.37261	58.44396	0
35.34214	58.28109	0
35.34535	58.23278	0
35.37181	58.21523	0
35.36889	58.25293	0
35.37736	58.28091	0
35.44377	58.30102	0
35.45778	58.35392	0
35.44928	58.35984	0
35.3961	58.4318	0
35.26463	59.14178	0
35.25627	59.15114	0
35.26127	59.17111	0
35.26075	59.18886	0
35.24403	59.19978	0
35.23368	59.19235	0
35.23866	59.15601	0
35.29713	59.12871	0
35.28699	59.11284	0
35.28015	59.09364	0
35.277	59.07838	0
35.24984	59.08443	0
35.2402	59.10277	0
35.23512	59.17875	0
35.25525	59.13313	0
35.25843	59.1142	0
35.26416	59.12578	0
36.33332	57.68255	0
36.33255	57.69632	0
36.32304	57.6829	0
36.32548	57.70544	0
36.33794	57.71339	0
36.34203	57.72872	0
36.35187	57.75715	0
36.36527	57.69769	0
36.36868	57.67835	0
36.37568	57.63086	0
36.38151	57.60539	0
36.3969	57.6197	0
36.40404	57.69305	0

36.41902	57.65823	0
36.44731	57.60806	0
36.44786	57.56328	0
36.44262	57.65782	0
36.42585	57.4708	0
36.39843	57.43774	0
36.36726	57.3447	0
36.36341	57.31635	0
36.37589	57.27777	0
36.3914	57.25849	0
36.43148	57.25082	0
36.4605	57.26328	0
36.4649	57.3151	0
36.43913	57.21071	0
36.50859	57.18968	0
36.56405	57.16034	0
36.54136	57.04565	0
36.51588	56.99868	0
36.47904	56.96179	0
36.49078	56.91475	0
36.57191	56.9131	0
36.61887	56.97025	0
36.57903	56.88099	0
36.55428	56.83898	0
35.72115	59.53133	0
35.77661	59.54108	0
35.79855	59.50884	0
35.79207	59.44808	0
35.75983	59.41854	0
35.72512	59.41773	0
35.68589	59.41089	0
35.64245	59.70844	0
35.67901	59.69582	0
35.74908	59.625	0
35.72734	59.54931	0
35.71448	59.48427	0
35.68544	59.42804	0
35.68816	59.3947	0
35.69754	59.31648	0
35.71201	59.27765	0
35.7631	59.28819	0
35.7848	59.33468	0
35.79834	59.46351	0
35.8092	59.51564	0
35.80852	59.47539	0

35.77062	59.42239	0
35.7967	59.39654	0
35.77611	59.31988	0
35.80742	59.32285	0
35.74301	59.26012	0
35.71045	59.25266	0
35.1766	60.35201	0
35.19335	60.32427	0
35.21031	60.30375	0
35.22184	60.305	0
35.21815	60.28941	0
35.21178	60.28027	0
35.19999	60.28837	0
35.20178	60.31065	0
35.18414	60.33048	0
35.19181	60.35425	0
35.19832	60.36249	0
35.18962	60.37011	0
35.17668	60.36943	0
35.16564	60.36496	0
35.15447	60.35697	0
35.15479	60.37349	0
35.15592	60.39239	0
35.14816	60.40849	0
35.13933	60.39025	0
35.13816	60.37323	0
35.12848	60.41686	0
35.11789	60.41428	0
35.10804	60.40233	0
35.09698	60.42295	0
35.08108	60.43692	0
35.06995	60.44436	0
36.46553	59.20143	0
36.45062	59.18848	0
36.44226	59.172	0
36.42833	59.15121	0
36.42427	59.16172	0
36.41895	59.14681	0
36.29559	59.6452	0
36.29335	59.06367	0
36.28337	59.05027	0

decimalLatitude	decimalLongitude	Euphorbia.ferdowsiana
36.345	59.055	1
36.28	59.124	1
36.316	59.202	1
36.30516	59.07874	1
36.30613	59.0787	1
36.087	59.288	0
36.121	59.37	0
36.274	59.116	0
36.305	59.155	0
36.354	58.889	0
36.377	59.258	0
36.584	58.949	0
36.59657	58.59321	0
36.78083	58.55241	0
36.36046	59.11382	0
36.3639	59.11003	0
36.72587	59.84027	0
36.68079	59.92821	0
36.69714	59.83772	0
36.6933	59.79366	0
36.69085	59.77002	0
36.75945	59.87055	0
36.7865	59.88192	0
36.78007	59.84174	0
36.74736	59.77523	0
36.77661	59.80681	0
36.75023	59.74981	0
36.66066	59.61685	0
36.69443	59.63573	0
36.80957	59.85363	0
36.79735	59.80603	0
36.78855	59.71586	0
36.78053	59.64075	0
36.76374	59.60461	0
36.75225	59.5694	0
36.70963	59.51126	0
36.73656	59.54857	0
36.74391	59.55933	0
36.76672	59.56239	0
36.84574	59.59843	0
36.83167	59.57944	0
36.90834	59.74107	0
36.88123	59.69605	0
36.86461	59.6513	0

36.85712	59.62217	0
36.81668	59.54302	0
36.81346	59.4931	0
36.96459	59.71897	0
36.91806	59.6534	0
36.90367	59.61714	0
36.89321	59.5878	0
36.87908	59.55394	0
36.8637	59.53741	0
36.84516	59.52282	0
36.82904	59.50353	0
36.80304	59.48163	0
36.77382	59.42939	0
36.80013	59.45515	0
36.82079	59.48514	0
36.84604	59.50428	0
36.88925	59.55656	0
36.91446	59.57303	0
36.93449	59.60411	0
36.93781	59.54898	0
36.91781	59.52216	0
36.9052	59.49514	0
36.89924	59.48142	0
36.89694	59.46765	0
36.87993	59.44947	0
36.86421	59.43076	0
36.85082	59.4125	0
36.82932	59.39336	0
36.83251	59.36401	0
36.85704	59.37502	0
36.87798	59.40154	0
36.88912	59.41974	0
36.90673	59.42252	0
36.92152	59.44999	0
36.93699	59.45983	0
36.97345	59.48022	0
36.99489	59.51211	0
37.02616	59.53403	0
36.99658	59.47834	0
36.97833	59.42321	0
36.97149	59.40169	0
36.95078	59.38326	0
36.93573	59.35447	0
36.91037	59.3211	0
36.90191	59.31593	0

36.92895	59.32427	0
36.84723	59.28712	0
36.89299	59.30308	0
36.93543	59.31138	0
37.00958	59.38496	0
37.04352	59.41232	0
37.03285	59.37978	0
37.00578	59.32974	0
37.00474	59.28308	0
37.02931	59.30033	0
37.03428	59.24338	0
37.07683	59.1004	0
36.9933	59.01839	0
37.05623	59.05242	0
37.05192	58.97054	0
36.97395	58.78465	0
37.03526	58.78311	0
37.09076	58.71078	0
36.71629	60.23407	0
36.71048	60.18659	0
36.69877	60.12353	0
36.67315	60.02527	0
36.65667	59.96075	0
36.63567	59.91268	0
36.57297	59.84025	0
36.52198	59.76838	0
36.47429	59.64499	0
36.45121	59.5874	0
36.38078	59.63273	0
36.38885	59.76544	0
36.31551	59.77293	0
36.39125	59.63537	0
36.78287	59.9055	0
36.84537	59.86076	0
36.84802	59.79867	0
36.834	59.75032	0
36.68648	59.67176	0
36.97482	59.64401	0
36.96569	59.61726	0
36.9428	59.58962	0
36.92633	59.57685	0
36.75098	59.00311	0
36.72546	58.99801	0
36.69726	59.0388	0
36.6704	59.04602	0

36.63369	59.06737	0
36.60689	59.08867	0
36.60017	59.27708	0
36.50842	59.46393	0
36.57348	60.02637	0
36.62673	60.04379	0
36.63979	59.957	0
36.64006	59.90089	0
36.69205	59.9505	0
36.70589	60.06947	0
36.74097	60.1353	0
36.78801	59.89403	0
36.79279	59.83774	0
36.78842	59.7974	0
36.77461	59.67137	0
36.80672	59.65334	0
36.76058	59.63706	0
36.72617	59.61535	0
36.71188	59.60217	0
36.69208	59.5939	0
36.75444	59.61189	0
36.76461	59.51963	0
36.74641	59.46525	0
36.73929	59.41904	0
36.74317	59.37281	0
36.70056	59.32138	0
36.68304	59.29178	0
36.64842	59.3232	0
37.10519	58.66687	0
37.16153	58.74415	0
37.39725	58.75718	0
37.42973	58.76681	0
37.39064	58.7024	0
37.36887	58.72768	0
37.40603	58.80474	0
37.42279	58.85227	0
37.3962	58.8224	0
37.35538	58.79043	0
37.34913	58.7529	0
37.3335	58.79561	0
37.28931	58.7534	0
37.2315	58.74865	0
37.17178	58.78164	0
37.18623	58.89712	0
37.24655	58.96168	0

37.18451	58.93679	0
37.23935	59.12079	0
37.22348	59.20629	0
37.17744	59.21417	0
37.10345	59.18024	0
37.06172	59.13421	0
37.10752	59.30888	0
37.10749	59.38991	0
36.87114	59.00148	0
36.94362	59.11303	0
36.89063	59.04438	0
36.95862	59.34409	0
36.97345	59.56467	0
36.91957	59.62678	0
36.85793	59.6341	0
36.77211	59.70606	0
36.74705	59.90592	0
36.64895	59.88526	0
37.06202	59.51236	0
37.08206	59.72071	0
37.09632	59.68558	0
36.87275	59.94283	0
36.84491	59.90472	0
36.74312	59.83154	0
36.68538	59.78646	0
36.63545	59.74637	0
36.65659	59.72264	0
36.63778	60.00589	0
36.63992	60.06291	0
36.6188	60.05853	0
36.58251	60.06474	0
36.35421	59.95143	0
36.41766	59.79367	0
36.63132	59.94818	0
36.64742	60.13899	0
35.91736	59.90191	0
37.13383	59.18624	0
37.11954	59.17257	0
37.12809	59.24688	0
37.10541	59.23697	0
37.07331	59.24598	0
37.13301	59.28483	0
37.10573	59.33741	0
37.08276	59.39067	0
37.05193	59.4345	0

37.03696	59.4313	0
37.0189	59.42701	0
37.01009	59.41643	0
36.99706	59.41325	0
36.98784	59.42173	0
37.01316	59.46277	0
37.04119	59.48855	0
37.0488	59.52081	0
37.03964	59.54007	0
37.0267	59.55947	0
37.58387	58.50859	0
37.58718	58.4829	0
37.59046	58.46525	0
37.61098	58.45875	0
37.6278	58.44982	0
37.6236	58.4209	0
37.62893	58.40728	0
37.63828	58.40899	0
37.6429	58.42152	0
37.63547	58.43907	0
37.64031	58.46109	0
37.63324	58.46883	0
37.62705	58.49811	0
37.56229	58.5277	0
37.55375	58.51759	0
37.54309	58.52863	0
37.51992	58.50506	0
37.50339	58.50401	0
37.49213	58.49729	0
37.47556	58.51304	0
37.48657	58.56216	0
37.46334	58.52748	0
37.4475	58.54779	0
37.44398	58.57374	0
37.46805	58.60307	0
37.49332	58.62248	0
37.5142	58.65011	0
37.57022	58.64852	0
37.58447	58.6235	0
37.57974	58.64947	0
37.59351	58.6549	0
37.56914	58.67166	0
37.5553	58.6723	0
37.53933	58.66175	0
37.52176	58.66365	0

37.50316	58.66087	0
37.48358	58.64729	0
37.46868	58.64103	0
37.45845	58.62539	0
37.44518	58.61833	0
37.43593	58.61947	0
37.43367	58.63367	0
37.44385	58.66118	0
37.43381	58.68178	0
37.44496	58.70646	0
37.46636	58.7246	0
37.46565	58.7445	0
37.47395	58.76965	0
37.48082	58.78849	0
37.47879	58.80368	0
37.47429	58.81453	0
37.4612	58.81204	0
37.4419	58.81486	0
37.42987	58.81314	0
37.41757	58.80764	0
37.40642	58.79225	0
37.3968	58.78119	0
37.38725	58.78461	0
37.37434	58.78114	0
37.36193	58.78201	0
37.35337	58.7765	0
37.33344	58.77286	0
37.31762	58.76875	0
37.3056	58.76627	0
37.29588	58.76719	0
37.28593	58.77233	0
37.28059	58.79659	0
37.27048	58.78714	0
37.27333	58.80388	0
37.27126	58.82509	0
37.26279	58.82949	0
37.25796	58.83984	0
37.27524	58.87566	0
37.29247	58.90336	0
37.2905	58.93204	0
37.31318	58.99175	0
37.30367	58.99031	0
37.28865	58.99512	0
37.27169	58.99948	0
37.25487	58.99908	0

37.28125	59.03889	0
37.26863	59.03595	0
37.2791	59.07253	0
37.26085	59.07753	0
37.24458	59.07515	0
37.22337	59.07305	0
37.19895	59.03243	0
37.23969	59.08601	0
37.2451	59.10678	0
37.22358	59.11034	0
37.20734	59.12431	0
37.19077	59.12379	0
37.16716	59.10816	0
37.14709	59.08275	0
37.12363	59.0788	0
37.09724	59.14135	0
37.15932	59.22035	0
36.98804	59.36854	0
36.97724	59.34903	0
36.96822	59.35293	0
36.97573	59.36268	0
36.97308	59.37416	0
36.96393	59.37963	0
36.97198	59.38929	0
36.95978	59.39166	0
36.9489	59.46336	0
36.94674	59.52436	0
36.90193	59.52708	0
36.86525	59.51955	0
36.84431	59.4805	0
36.83997	59.40608	0
36.80984	59.39116	0
36.77979	59.36932	0
36.78391	59.44785	0
36.79904	59.51969	0
36.81921	59.56883	0
36.83278	59.61224	0
36.86601	59.66622	0
36.89855	59.73299	0
36.90942	59.70175	0
36.87875	59.73941	0
36.86591	59.75986	0
36.85629	59.77853	0
36.83315	59.76618	0
36.805	59.75915	0

36.78632	59.75588	0
36.76819	59.75624	0
36.73565	59.7701	0
36.70669	59.7554	0
36.6754	59.7704	0
36.66928	59.74067	0
36.69311	59.98263	0
36.71901	60.01837	0
36.72071	59.81109	0
36.69962	59.77564	0
36.66905	59.80517	0
36.6202	59.75571	0
36.41803	60.19444	0
36.46594	60.23577	0
36.47342	60.10972	0
36.50448	60.07126	0
36.51629	60.09728	0
36.21894	60.23183	0
36.67597	59.88319	0
36.7147	59.86797	0
36.78502	59.86394	0
36.51202	59.76094	0
36.46622	59.81054	0
36.40607	59.84376	0
36.31713	59.85008	0
36.26437	59.92923	0
36.29456	59.6812	0
36.36691	59.59197	0
36.45213	59.54145	0
36.54269	59.40892	0
36.53261	59.3147	0
36.54833	59.24238	0
37.02681	58.74645	0
36.98371	58.65318	0
36.96409	58.56462	0
37.08635	58.52685	0
37.1551	58.58764	0
37.52053	58.58357	0
37.5915	58.58622	0
37.48382	58.71888	0
37.72075	58.31453	0
37.68569	58.24729	0
37.64897	58.30177	0
37.61491	58.37028	0
37.54925	58.43399	0

37.4854	58.48926	0
37.33888	58.84272	0
37.24313	58.99892	0
37.14985	58.97217	0
37.69238	58.43288	0
37.76501	58.26385	0
37.80498	58.14663	0
37.47866	58.175	0
36.60172	64.29742	0
34.46007	63.91618	0
34.45606	63.62844	0
34.4866	63.38501	0
34.4599	63.10192	0
34.44382	62.89625	0
34.62113	62.58306	0
34.63376	62.37054	0
33.8914	62.6445	0
33.63138	62.63398	0
33.43861	63.03738	0
34.86632	60.1907	0
35.04577	59.89041	0
35.51433	59.84767	0
35.38658	58.65703	0
33.35841	58.48966	0
35.63335	58.52979	0
35.52552	58.77781	0
36.58241	54.35698	0
36.63097	54.66044	0
36.66189	54.86709	0
36.76782	55.20019	0
37.05097	55.70175	0
37.37109	56.09588	0
37.45043	56.41716	0
38.80722	57.7982	0
37.82522	58.07355	0
37.35539	54.4676	0
37.39443	56.73825	0
35.4077	58.27722	0
35.36968	58.51215	0
35.3624	58.52906	0
35.34902	58.54431	0
35.33078	58.54551	0
35.31905	58.5733	0
35.33318	58.60281	0
35.34986	58.61669	0

35.36353	58.56574	0
35.36217	58.65041	0
35.34526	58.64843	0
35.33326	58.6522	0
35.35794	58.75499	0
35.33701	58.8087	0
35.36717	58.72784	0
35.37657	58.67398	0
35.37077	58.58624	0
35.35738	58.48484	0
35.37261	58.44396	0
35.34214	58.28109	0
35.34535	58.23278	0
35.37181	58.21523	0
35.36889	58.25293	0
35.37736	58.28091	0
35.44377	58.30102	0
35.45778	58.35392	0
35.44928	58.35984	0
35.3961	58.4318	0
35.26463	59.14178	0
35.25627	59.15114	0
35.26127	59.17111	0
35.26075	59.18886	0
35.24403	59.19978	0
35.23368	59.19235	0
35.23866	59.15601	0
35.29713	59.12871	0
35.28699	59.11284	0
35.28015	59.09364	0
35.277	59.07838	0
35.24984	59.08443	0
35.2402	59.10277	0
35.23512	59.17875	0
35.25525	59.13313	0
35.25843	59.1142	0
35.26416	59.12578	0
36.33332	57.68255	0
36.33255	57.69632	0
36.32304	57.6829	0
36.32548	57.70544	0
36.33794	57.71339	0
36.34203	57.72872	0
36.35187	57.75715	0
36.36527	57.69769	0

36.36868	57.67835	0
36.37568	57.63086	0
36.38151	57.60539	0
36.3969	57.6197	0
36.40404	57.69305	0
36.41902	57.65823	0
36.44731	57.60806	0
36.44786	57.56328	0
36.44262	57.65782	0
36.42585	57.4708	0
36.39843	57.43774	0
36.36726	57.3447	0
36.36341	57.31635	0
36.37589	57.27777	0
36.3914	57.25849	0
36.43148	57.25082	0
36.4605	57.26328	0
36.4649	57.3151	0
36.43913	57.21071	0
36.50859	57.18968	0
36.56405	57.16034	0
36.54136	57.04565	0
36.51588	56.99868	0
36.47904	56.96179	0
36.49078	56.91475	0
36.57191	56.9131	0
36.61887	56.97025	0
36.57903	56.88099	0
36.55428	56.83898	0
35.72115	59.53133	0
35.77661	59.54108	0
35.79855	59.50884	0
35.79207	59.44808	0
35.75983	59.41854	0
35.72512	59.41773	0
35.68589	59.41089	0
35.64245	59.70844	0
35.67901	59.69582	0
35.74908	59.625	0
35.72734	59.54931	0
35.71448	59.48427	0
35.68544	59.42804	0
35.68816	59.3947	0
35.69754	59.31648	0
35.71201	59.27765	0

35.7631	59.28819	0
35.7848	59.33468	0
35.79834	59.46351	0
35.8092	59.51564	0
35.80852	59.47539	0
35.77062	59.42239	0
35.7967	59.39654	0
35.77611	59.31988	0
35.80742	59.32285	0
35.74301	59.26012	0
35.71045	59.25266	0
35.1766	60.35201	0
35.19335	60.32427	0
35.21031	60.30375	0
35.22184	60.305	0
35.21815	60.28941	0
35.21178	60.28027	0
35.19999	60.28837	0
35.20178	60.31065	0
35.18414	60.33048	0
35.19181	60.35425	0
35.19832	60.36249	0
35.18962	60.37011	0
35.17668	60.36943	0
35.16564	60.36496	0
35.15447	60.35697	0
35.15479	60.37349	0
35.15592	60.39239	0
35.14816	60.40849	0
35.13933	60.39025	0
35.13816	60.37323	0
35.12848	60.41686	0
35.11789	60.41428	0
35.10804	60.40233	0
35.09698	60.42295	0
35.08108	60.43692	0
35.06995	60.44436	0
36.46553	59.20143	0
36.45062	59.18848	0
36.44226	59.172	0
36.42833	59.15121	0
36.42427	59.16172	0
36.41895	59.14681	0
36.29559	59.6452	0
36.29335	59.06367	0

36.28337	59.05027	0
