# Supplementary material for: Predicting range shifts of three endangered endemic plants of the Khorassan-Kopet Dagh floristic province under global change
Source: Sci Rep. 2021 Apr 28;11:9159. doi: 10.1038/s41598-021-88577-x (PMC8080812; doi:10.1038/s41598-021-88577-x)
Supplement: Supplementary file 2 — Supplementary Information 2. [file 41598_2021_88577_MOESM2_ESM.docx]

Predicting range shifts of three endangered endemic plants of the Khorassan-Kopet Dagh floristic province under global change

Mohammad Bagher Erfanian^1,*^, Mostafa Sagharyan^2^, Farshid Memariani^3^, and Hamid Ejtehadi^1^

^1^ *Quantitative Plant Ecology and Biodiversity Research Lab., Department of Biology, Faculty*

*of Science, Ferdowsi University of Mashhad, Mashhad, Iran*

^2^ *Department of Plant Biology, Faculty of Biological Science, Tarbiat Modares University, Tehran, Iran*

^3^ *Department of Botany, Research Center for Plant Sciences, Ferdowsi University of Mashhad, Mashhad, Iran*

^*^Corresponding author: M. B. Erfanian, [b.erfanian@um.ac.ir](mailto:b.erfanian@um.ac.ir), Tel.: +98-51-38804167, Fax: +98-51-38796416, PO BOX 9177948974

Table S1- The selected variables for species distribution modelling of three studies species. Importance is reported for the final ensemble model.

| *Nepeta binaloudensis* | | *Phlomoides binaludensis* | | *Euphorbia ferdowsiana* | |
| --- | --- | --- | --- | --- | --- |
| Variable name | Importance | Variable name | Importance | Variable name | Importance |
| BIO5 | 0.48 | BIO7 | 0.15 | BIO5 | 0.57 |
| BIO8 | 0.04 | BIO8 | 0.02 | BIO8 | 0.06 |
| BIO18 | 0.12 | BIO10 | 0.38 | BIO18 | 0.09 |
| BIO19 | 0.32 | BIO18 | 0.03 | BIO19 | 0.18 |
| Elevation | 0.04 | BIO19 | 0.39 | Elevation | 0.10 |
| ** | ** | Elevation | 0.03 | ** | ** |
